# Proteomic Analysis of Differential Expression of Cellular Proteins in Response to Avian H9N2 Virus Infection of A549 Cells

**DOI:** 10.3389/fmicb.2016.01962

**Published:** 2016-12-15

**Authors:** Guanliu Yu, Wei Liang, Jiyuan Liu, Dan Meng, Liangmeng Wei, Tongjie Chai, Yumei Cai

**Affiliations:** College of Veterinary Medicine, Shandong Agricultural University, Sino-German Cooperative Research Centre for Zoonosis of Animal Origin Shandong ProvinceTai’an, China

**Keywords:** H9N2 AIV, two-dimensional electrophoresis, A549 cells, differentially expressed proteins, proteomics

## Abstract

In this study, differentially expressed proteins in A549 cells (human lung adenocarcinoma epithelial cell line) infected with H9N2 avian influenza virus (AIV) were investigated by two-dimensional electrophoresis (2-DE). Sixteen different spots between the groups (ratio > 2, *p* < 0.05) were identified with mass spectrometry identification. Proteins located in the downstream of the NF-κB and IFN transcription factor pathways were identified, e.g., ISG15. Actin and keratin were also identified, suggesting that the cytoskeleton may plays an important role in the AIV infection of mammalian cells. These findings could provide insights into the interaction between host and influenza viruses and might provide valuable information for clarifying the pathogenesis of viral infections as well.

## Introduction

The avian influenza virus (AIV) H9N2 subtype has been circulating in domestic poultry in mainland China since 1994 (Tang et al., 1998). H9N2 influenza viruses occasionally transmit to humans, which have raised public concerns about a pandemic potential for this subtype of influenza viruses (Lin et al., 2000; Matrosovich et al., 2001). The infection of humans with avian H9N2 virus has been reported in China since the late 1990s (Peiris et al., 1999; Butt et al., 2005; Cheng et al., 2011). Some isolates of H9N2 viruses with the ability to binding SA-a2,6 receptors can infect humans (Ha et al., 2001; Matrosovich et al., 2001). The expanded receptor specificity of H9N2 AIVs has raised concerns about their pathogenicity in humans (Huang et al., 2014). Recently, increasing evidence has shown that avian H9N2 virus might act as a source of novel human influenza viruses (Jin et al., 2014). The internal genes of newly emerged human infections with AIV subtype H7N9 and H10N8 subtype AIV are derived from H9N2 AIV (Liu et al., 2013, 2015). There have been a lot of relevant reports about the mechanism underlying the pathogenicity and transmission of H9N2 AIV genes (Li et al., 2012, 2014; Zhong et al., 2014). However, there have been fewer proteomic studies on the host after infection (Liu et al., 2008). Therefore, there is a growing need to investigate host cells infected with the H9N2 virus to elucidate potential target proteins for viral infection and adaptation studies.

Researchers have applied proteomic approaches to study cellular proteins involved in the process of H5N1, H3N2, and H1N1 virus infection (Baas et al., 2006; Mayer et al., 2007; Coombs et al., 2010; Wu et al., 2013). However, alterations of cellular proteins in human airway epithelial cell lines infected by H9N2 influenza virus have not been reported. H9N2 AIV can infect the target cells of human lung tissue, namely type II alveolar cells and bronchial epithelial cells (Zhang et al., 2013). The A549 cell line, which originated from human airway epithelial cells, was found susceptible to a strain of H9N2 influenza viruses that we screened (unpublished data). In addition to this, A549 cell line has been used for H9N2 *in vitro* studies (Lee et al., 2010; Shahsavandi et al., 2013; Hamidreza et al., 2016). Therefore, the A549 cell line was selected for the present proteomic study.

To better understand the molecular and cellular basis of H9N2 infection and adaptation in human airway cells, we used proteomic approaches to study the patterns of cellular proteins with variable expression upon H9N2 virus infection. Our findings may assist with the investigations into the pathogenesis and adaptation of H9N2 virus in airway epithelial cells, and with the search for potential protein targets for further studies.

## Materials and Methods

### Cell Culture and Infection

Human lung epithelial A549 cells (ATCC CRL-185^TM^) were cultured in Dulbecco’s Modified Eagle’s medium (DMEM) (GIBCO, Grand Island, NY, USA) at pH 7.2, supplemented with 10% fetal bovine serum (TransGen, Beijing, China) and penicillin (100 U/mL)/streptomycin (100 μg/mL) and were grown in an incubator at 37°C in a 5% CO_2_ humidified atmosphere.

In order to examine the difference of expressed proteins of the H9N2 virus in A549 cells effectively, specific pathogen free (SPF) embryonic chicken eggs (9-day-old) were used to amplify H9N2 virus - A/Chicken/Shandong/ch/2011(CK/SD/ch), which provided by the Center for Animal Disease Control Engineering of Shandong Province. Next, the amplified H9N2 virus was inoculated onto monolayers of the A549 cells line (1.6 × 10^6^ cells/mL), a multiplicity of infection (MOI) of 1 was used in this study. Viral infection was carried out in DMEM with 2% FBS and pen/strep at 37°C in 5% CO_2_. The control groups were infected with the allantoic fluid of a healthy SPF chick embryo. The infected cells were harvested at 24 h post-infection (hpi) and 72 hpi by scraping followed by washing three times with 1 × PBS. In addition, in order to measure the titer of H9N2 virus, a standard hemagglutination (HA) titer assay was conducted.

### Sample Preparation

The cells were lysed with a lysis buffer containing 7 M urea, 2 M thiourea, 4% w/v CHAPS, 40 mM DTT, and 2% v/v IPG Buffer pH 4-7. After 60 min of gentle stirring at room temperature, the sample was centrifuged at 18 000 ×*g* at 4°C for 60 min. The supernatant was collected and the protein concentration was determined using the Bradford protein assay kit (TIANGEN, Beijing, China), according to the manufacturer’s instructions. The samples were then aliquoted and stored at -80°C until subsequent use.

### Indirect Immunofluorescence

A549 cells were fixed with an acetone and ethanol solution (acetone: ethanol D 3:2) for 5 min, then washed with phosphate-buffered saline (PBS). Air-dried cells were incubated with an anti-hemagglutinin (HA) monoclonal antibody at a 1:500 dilution, at 37°C, for 1 h. After three washes with PBS, cells were incubated with fluorescein isothiocyanate (FITC)-conjugated goat anti-mouse IgG at a 1:500 dilution, at 37°, for 45 min. After three washes with PBS, H9N2 virus infected cells were air dried, and visualized and imaged with a Nikon inverted fluorescence microscope.

### Two-Dimensional Gel Electrophoresis (2-DE)

Isoelectric focusing was carried out for a 1 h step-and-hold at 500 V, 1 h at a 1000 V gradient, 3 h at an 8000 V gradient, and a 2.5 h step-and-hold at 8000 V. After equilibration, strips were loaded on SDS–PAGE gels, and were electrophoresed for 45 min at a power of 5 W per gel, and then for approximately 6 h at a power of 12 W per gel until the dye reached the bottom of the gels. After electrophoresis, the gels were stained using silver staining methods.

### Protein Identification and Database Search

The stained gels were scanned with Imagescanner III, and the gel images were processed by Image Master 2D Platinum 7.0 software. The differentially expressed protein spots (*p* < 0.05) with at least a two-fold difference in intensity were selected and subject to identification. Interesting protein spots were picked out from the stained gels, subjected to in-gel tryptic digestion and subsequently subjected to identification by Matrix-assisted Laser Desorption/Ionization Time-of-Flight Mass Spectrometry (MALDI-TOF/TOF) analysis. Combined mass spectrometry (MS) and tandem mass spectrometry (MS/MS) queries were performed using the Mascot search engine 2.2 in the NCBI-HUMAN database (268811, August 30, 2013) and uniprot-*Homo sapiens* database (134919, December 24, 2013).

## Results

In current study, we examined the HA titer of H9N2 before infection, and the titer was 2^7^. However, the titers in supernatant of infected A549 cells were 2^8^ (24 hpi) and 2^4^ (72 hpi), respectively. The changes characteristic to the type of cytopathic effect of A549 cells following 24 hpi and 72 hpi with light microscope. The cells had minimal changes, mainly in cell shrinkage, rounding and suspension of a few of them at 24 hpi and about half lost the adhesion to the surface of the cell culture plastic at 72 hpi.

**FIGURE 1:**
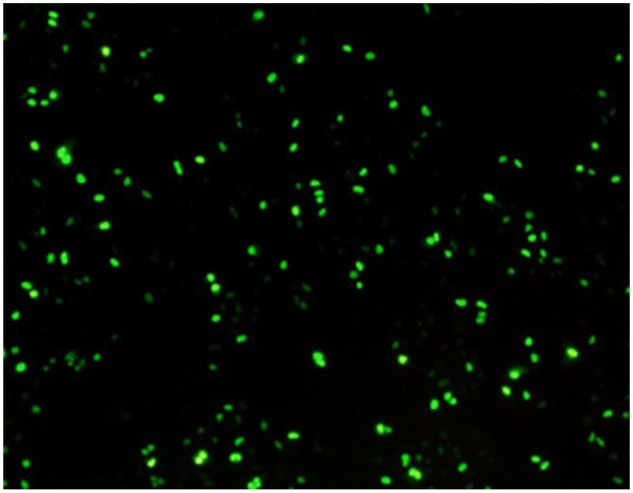
**Indirect immunofluorescence analysis of A549 cells infected with CK/SD/ch confirmed infection.** (Approximately 45% of the cells were infected by H9N2 at 24 hpi).

Next, indirect immunofluorescence assay (IFA) was used to verify the infection of A549 cells by H9N2 AIV. Mouse monoclonal antibody against viral nucleoprotein was used as the primary antibody and goat antimouse IgG/FITC was the secondary antibody. The result showed that about 45% of the A549 cells were infected by CK/SD/ch at 24 hpi (**Figure 1**).

**FIGURE 2:**
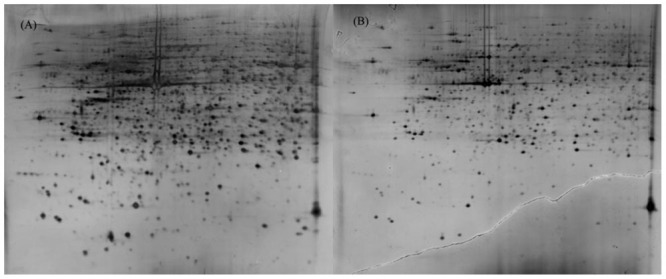
**2-DE gel images of AIV-infected and control groups of A549 cells at 24 hpi. (A)**: 2-DE gel of the AIV-infected group. **(B)**: 2-DE gel of the control group.

**FIGURE 3:**
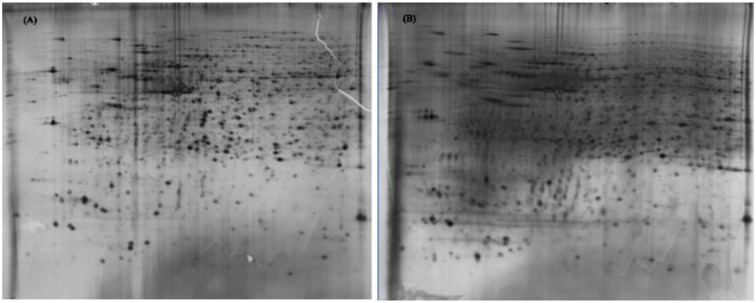
**2-DE gel images of AIV-infected and control groups of A549 cells at 72 hpi. (A)**: 2-DE gel of the AIV-infected group. **(B)**: 2-DE gel of the control group.

**FIGURE 4:**
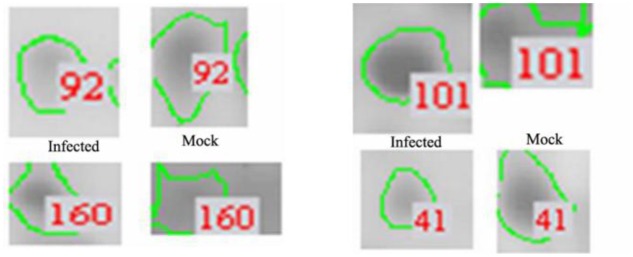
**Enlarged regions of several differentially expressed protein spots.** Differentially expressed protein spots are indicated by numbers and circles.

Protein extracts (300 μg) from A549 cells at different time post infection were loaded on to 2-DE gels. For each time point three replicate gels were run. A total of 481 proteins were detected in every gel. Differentially expressed proteins were judged by the criterion of an increase or decrease of spot intensities by at least two fold (**Figures 2–3**). The majority of the differentially expressed protein spots were also illustrated in enlarged formats (**Figure 4**). After searching NCBI-HUMAN and uniprot**-***Homo sapiens* databases using the Mascot search engine, sixteen differentially expressed proteins were identified at different time points post-infection. Six protein spots of them (spots 319, 194, 167, 166, 160, and 9) were found to be up-regulated at both 24 hpi and 72 hpi (**Tables 1** and **2**). Only three protein spots (spots 169, 92, and 90) were found to be down-regulated at above two time points (**Tables 3** and **4**).

**Table 1:** Summary of upregulated proteins in A549 cells infected with influenza A H9N2 virus at 24 hpi (*r* > 2, *p* < 0.05).

Spot ID	Protein information	Accesssion no.	Protein MW(Da)	Protein PI	Function
150	Actin, cytoplasmic 1	gi|148231177	42081.9	5.30	Cytoskeleton protein
160	Complement component 1 Q subcomponent-binding protein, mitochondrial precursor	gi|4502491	31741.8	4.73	Protein binding activity
166	Keratin 10	gi|186629	39832.1	4.72	Cytoskeleton protein
9	Heat shock factor protein 4	gi|100913209	53010.63	5.29	Regulation of transcription from RNA polymerase II promoter
194	HUMAN Complement C3	gi|78101267	187145.88	6.02	Inflammatory response
319	HUMAN Complement C1q tumor necrosis factor-related protein 5	gi|14149712	25298.1	6.05	Collagen trimer
167	HUMAN Lactotransferrin	gi|253723186	78181.12	8.5	Humoral immune response
86	HUMAN Apolipoprotein A-I	gi|2914176	30777.44	5.56	Negative regulation of interleukin-1 beta secretion
101	HUMAN L-lactate dehydrogenase B chain	gi|13786848	36638.07	5.71	Extracellular vesicular exosome

**Table 2:** Summary of upregulated proteins in A549 cells infected with influenza A H9N2 virus at 72 hpi (*r* > 2, *p* < 0.05).

Spot ID	Protein information	Accesssion no.	Protein MW(Da)	Protein PI	Function
160	Complement component 1 Q subcomponent-binding protein, mitochondrial precursor	gi|4502491	31741.8	4.73	Protein binding activity
166	Keratin 10	gi|186629	39832.1	4.72	Cytoskeleton protein
9	Heat shock factor protein 4	gi|100913209	53010.63	5.29	Regulation of transcription from RNA polymerase II promoter
194	HUMAN Complement C3	gi|78101267	187145.88	6.02	Inflammatory response
319	HUMAN Complement C1q tumor necrosis factor-related protein 5	gi|14149712	25298.1	6.05	Collagen trimer
167	HUMAN Lactotransferrin	gi|119585171	78181.12	8.5	Humoral immune response
364	IFN-induced GTP-binding protein	gi|251757499	75877.3	5.60	Cytokine-mediated signaling pathway
434	Ubiquitin-like protein 15	gi|461287	17931.8	6.84	Defense response to virus
308	Cytokine-induced apoptosis inhibitor 1	gi|45501191	32823.5	5.55	Negative regulation of apoptotic process

**Table 3:** Summary of downregulated proteins in A549 cells infected with influenza A H9N2 virus at 24 hpi (*r* > 2, *p* < 0.05).

Spot ID	Protein information	Accesssion no.	Protein MW(Da)	Protein PI	Function
90	HnRNP U	gi|14141161	83776.5	6.29	RNA processing
92	Selenide, water dikinase 1	gi|24797148	43390.7	5.90	Cellular protein modification process
169	Isoform 4 of hnRNPs C1/C2	gi|117189975	27845.2	5.75	RNA processing

**Table 4:** Summary of downregulated proteins in A549 cells infected with influenza A H9N2 virus at 72 hpi (*r* > 2, *p* < 0.05).

Spot ID	Protein information	Accesssion no.	Protein MW(Da)	Protein PI	Function
90	HnRNP U	gi|14141161	83776.5	6.29	RNA processing
92	Selenide, water dikinase 1	gi|24797148	43390.7	5.90	Cellular protein modification process
169	Isoform 4 of hnRNPs C1/C2	gi|117189975	27845.2	5.75	RNA processing
41	Isoform 1 of heat shock cognate 71-kDa protein	gi|5729877	71089.4	5.48	Regulation of cell cycle
521	Tubulin alpha-1B	gi|57013276	50805.2	5.43	Cellular response to interleukin-4

## Discussion

In this study, 24 h was selected as the first time point to carry out the statistics of differentially expressed proteins, for host cell morphology changes were not obvious at this time point. The differential expression proteins could affect response of antiviral immune of the host cells at this time point (Huang et al., 2014). 72 h was selected as the final stage of the infection to carry out statistics of differentially expressed proteins. Differential expression of proteins induced by human cells infected by AIV at different time points mainly involved cytoskeleton, cytokine mediated signaling pathways, mRNA transcription and expression of regulation, etc. (Liu et al., 2008; Sutejo et al., 2012).

In the current study, an overview of the differentially expressed proteins in response to infection by the H9N2 AIV was obtained by comparing differences in the abundance of proteins isolated from AIV-infected and mock-infected A549 cells.

Spot 150 (upregulated) was identified as actin, which is a major component of the cytoskeleton, and plays an important role in signaling pathways activated by virus infections (Liu et al., 2008). It is also thought to act as a regulator of transcription, for example, serve as a scaffold for the transport and/or anchorage of mRNA. The important roles of actin in gene transcription have been well described (Miralles and Visa, 2006). Actin can be used as a available transcription factor during virus synthetic protein using materials of host system. The actin cytoskeleton is also in favor of the release of progeny virus (Miralles and Visa, 2006). Besides, actin may also participate in endocytosis, by which the actin rearrangements contribute to virus particle internalization. This is achieved either by increasing endocytic activity (Pelkmans et al., 2002) or by bringing cell surface-bound virus particles to sites of high endocytic activity (Pollard and Borisy, 2003). The role of actin in H9N2 virus particle internalization in A549 cells needs further research.

Another component of cytoskeleton which displays considerable alteration in A549 cells infected with H9N2 influenza virus is cytokeretin, which forms intermediate filament in the cell. In this study, spot 166 (upregulated) was identified as keratin 10. In the cytoplasm, the network of cytokeratin filaments extending from the cell membrane to the nuclear membrane, play a role in the communication between the cell membrane and nuclear membrane. In addition, cytokeratin interacts with small nuclear ribonucleoprotein bodies containing small ribosomal protein and RNA.

In this study, actin and keratin in A549 cells infected with H9N2 AIV were identified, which may suggest that the cytoskeleton plays an important role in the AIV infection of mammalian cells with AIV.

Proteins located in the downstream of the NF-κB and IFN transcription factor pathways were identified, e.g., ISG15 (upregulated), which plays an important role in the antiviral process, especially that of the RNA virus. The ubiquitin-like modifier, ISG15, has been reported many times in the process of resisting influenza virus (Wu et al., 2013), and can be induced under virus and type I interferon stimulation. ISG15 plays an important role in regulating the antiviral innate immune response, and keeping interferon levels within a certain range, so that it can exert antiviral functions without causing immunologic damage. In addition, ISG15 may have antiviral effects by directly interacting with viral proteins (Zhao et al., 2010; Guan et al., 2011). In this study, it is possible that ISG15 was upregulated to play a role in the defense response to virus.

Spot 9 (downregulated) was HnRNP U (participating in pre-mRNA treatment) after identification, which interacts with NS1 protein during infection of H3N2 AIV (Coombs et al., 2010). NS1 protein can regulate cellular mRNA and viral mRNA translation. NS1 protein may hinder gene expression in normal cells by inhibiting ribonucleoprotein U-mediated mRNA expression. HnRNP U was downregulated in this study, which was consistent with previous findings during infections with H1N1 and H3N2 subtype viruses (Emmott et al., 2010). In our study, HnRNP U was also identified, which might suggest that HnRNP U can interact with NS1 during infection of H9N2 AIV.

To date, the antiviral role of some proteins involved in the anti-infection process of the host cell is ambiguous, therefore, we discussed the main differential proteins that were found in the current study. From the discussion above, actin, keratin, ISG15 and HnRNP U played an important role in the process of the A549 cells infected with H9N2 AIV. These findings could provide insights into the interaction between host and influenza viruses and may provide valuable information for clarifying the pathogenesis of viral infections.

## Author Contributions

GY and WL designed the experiment and completed most of the works. JL, DM, and LW analyzed some test results and collected materials. TC and YC gave experiment instruction. Thank all the authors’ contribution to the experiment.

## Conflict of Interest Statement

The authors declare that the research was conducted in the absence of any commercial or financial relationships that could be construed as a potential conflict of interest.
